# 10 Jahre Endoprothesenregister Deutschland (EPRD): was wurde erreicht?

**DOI:** 10.1007/s00132-023-04385-3

**Published:** 2023-05-23

**Authors:** Alexander W. Grimberg, Arnd Steinbrück

**Affiliations:** 1 EPRD Deutsche Endoprothesenregister gGmbH, Str. des 17. Juni 106–108, 10623 Berlin, Deutschland; 2 Orthopädisch-Chirurgisches Kompetenzzentrum Augsburg (OCKA), Augsburg, Deutschland

**Keywords:** Hüftendoprothese, Knieendoprothese, Implantatstandzeit, Qualitätssicherung, Datenanalyse, Hip arthroplasty, Knee arthroplasty, Implant survival, Quality assurance, Data analytics

## Abstract

Seit 10 Jahren erfasst und verfolgt das Endoprothesenregister Deutschland (EPRD) endoprothetische Eingriffe an Hüft- und Kniegelenk. Trotz der Freiwilligkeit des Registers konnten so bereits mehr als 2 Mio. Operationen in Deutschland erfasst werden. Das EPRD zählt somit als drittgrößtes Register der Welt. Die hochgranulare Klassifikation der EPRD-Produktdatenbank, in der mittlerweile mehr als 70.000 Artikel hinterlegt sind, soll zum internationalen Standard erhoben werden. Die Verknüpfung von Fällen mit spezifischen Implantatdaten sowie Routinedaten der Krankenkassen ermöglicht valide Standzeitanalysen. Auf dieser Basis erhalten Kliniken, Hersteller, aber auch die Fachöffentlichkeit spezifische Ergebnisse, die zur Verbesserung der Qualität in der Endoprothetik beitragen. Zunehmend internationale Wahrnehmung erhält das Register auch durch Publikationen in „peer-reviewed journals“. Ein Antragsverfahren ermöglicht zudem den Zugriff auf EPRD-Daten für „Dritte“. Darüber hinaus hat das EPRD ein Frühwarnsystem etabliert, um Auffälligkeiten zu detektieren. Softwaregestützt meldet das EPRD mögliche Fälle von Implantat-Mismatch an die betreffenden Kliniken. Die EPRD-Erfassung wird im Rahmen eines Probebetriebs im Jahr 2023 um Patientenbefragungen zur Zufriedenheit, sog. PROMs, ergänzt. Auch die Erfassung des Operateurs wird perspektivisch folgen.

## Abstract (en)

The German Arthroplasty Registry (EPRD) has been accumulating data on hip and knee arthroplasty procedures for 10 years. Even though the EPRD is a voluntary registry, it currently includes more than 2 million documented surgical procedures in Germany. This ranks the EPRD as the third largest registry in the world. The highly granular classification of the EPRD product database, which currently includes more than 70,000 components, is set to become the international standard. The linkage of hospital case data with specific implant component data and routine data from health insurance providers enables robust arthroplasty survival analyses. This provides hospitals, manufacturers, and the specialist community with access to specific results that contribute to improving arthroplasty quality. The registry is gaining increasing international recognition by publishing in peer-reviewed journals. An application procedure also allows “third-party” data access. Furthermore, the EPRD has also established an early warning system to detect anomalies in outcome. Software-based detection of potential implant component mismatches allows notification of hospitals concerned. In 2023, the EPRD will trial extending its data collection to include patient satisfaction surveys (i.e., patient-reported outcome measures), and subsequently to surgeon-specific data.

Endoprothesenregister haben in der Orthopädie eine mehr als 40-jährige Vorgeschichte, ihr Stellenwert für die Qualitäts- und Versorgungsforschung ist unbestritten. Mit seinem 10-jährigem Bestehen gehört das Endoprothesenregister Deutschland (EPRD) zu den jüngeren, aber auch moderneren Registern, die auf der Erfahrung der anderen aufbauen konnten. Dabei ist das EPRD eine freiwillige Initiative, die auch ohne eine gesetzliche Verpflichtung zur Teilnahme bereits viel erreicht hat.

## Hintergrund

Am 22. November 2012 war es endlich so weit. Im Rahmen des Probebetriebs wurden die ersten endoprothetischen Versorgungen an das Endoprothesenregister Deutschland (EPRD) übermittelt. Bis zum Ende des Probebetriebs am 30. Juni 2013 wurden 5620 Operationen an Hüft- und Kniegelenk aus insgesamt 28 Krankenhäusern dokumentiert [11]. Dass die Erfassung auf freiwilliger Basis und ohne entsprechende gesetzliche Anforderung gelang, lag maßgeblich an der engen Zusammenarbeit aller Beteiligten. Durch die Initiative der Deutschen Gesellschaft für Orthopädie und Orthopädische Chirurgie konnte gemeinsam mit dem AOK-Bundesverband GbR, dem Verband der Ersatzkassen e. V. (vdek) und den Endoprothesenherstellern, vertreten durch den Bundesverband Medizintechnologie e. V., die Grundlage dafür geschaffen werden, dass patientenbezogen prothesenspezifische Daten erhoben und diese mit Routinedaten der Kostenträger zusammengeführt werden konnten. Mit dieser strukturellen Besonderheit ließ sich der Erfassungsaufwand in den Krankenhäusern auf ein Minimum reduzieren. Der „datensparsame“ Ansatz war wesentlich für die initiale Akzeptanz der freiwillig teilnehmenden Kliniken. Mit Beginn der Datenerfassung stieg innerhalb weniger Jahre die Zahl der datenliefernden Kliniken kontinuierlich an und lag im Jahr 2021 bei 747 [9]. Gleichzeitig nahmen auch die Erfassungsraten innerhalb der Krankenhäuser zu. Durch das Engagement der Kliniken konnten so mittlerweile mehr als 2 Mio. Datensätze gesammelt werden [13]. Damit ist das EPRD nach dem britischen National Joint Registry (NJR) und dem US-amerikanischen American Joint Replacement Registry das drittgrößte Hüft- und Knieendoprothesenregister der Welt. Seit 2019 sammelt das EPRD mit über 300.000 Versorgungen pro Jahr – das entspricht einer Abdeckungsrate von ca. 70 % aller in Deutschland durchgeführten Eingriffe – mehr als jedes andere europäische Register [8].

### EPRD-Produktdatenbank

Eine weitere strukturelle Besonderheit des EPRD liegt in der Implantatproduktdatenbank, deren hoch-granulare Klassifikation gemeinsam mit den Endoprothesenherstellern entwickelt wurde. Die Verknüpfung von Patienten- und Versorgungsmerkmalen mit detaillierten, implantatspezifischen Informationen ermöglicht nicht nur die automatische Bestimmung von unterschiedlichen Versorgungsformen und eine Plausibilitätsprüfung der Datensätze, sondern auch die Evaluation des Einflusses unterschiedlicher Implantateigenschaften auf das Versorgungsergebnis in einer Detailtiefe, die international ihresgleichen sucht [1]. Auf Basis der ersten Version der EPRD-Produktdatenbank wurde die zugrunde liegende Klassifikation mit dem britischen NJR harmonisiert, sodass die Produktdatenbanken der beiden größten europäischen Register mittlerweile diese gemeinsame Einteilung nutzen, die – wenn es nach der International Society of Arthroplasty Registries geht, in der fast alle der weltweiten Endoprothesenregister vertreten sind – als globaler Standard fungieren soll.

### Nationale Abdeckungsrate vs. Vollständigkeit der Nachverfolgung

Die Freiwilligkeit des Registers hat aber auch ihren Preis. Das EPRD kann mittlerweile auf eine hohe nationale Abdeckung verweisen; dennoch können nicht alle Operationen lückenlos nachverfolgt werden. Das EPRD beschränkt sich bisher auf Versorgungen von gesetzlich versicherten Patienten mit Routinedaten aus dem AOK-Bundesverband oder dem vdek. Da diese beiden Verbände den überwiegenden Teil (ca. 75 %) aller gesetzlich Krankenversicherten in Deutschland repräsentieren [14], sind mittlerweile – trotz der genannten Einschränkung – über große Vergleichsgruppen hinweg valide Standzeitauswertungen möglich.

### Öffentliche Berichterstattung des Registers

Von Anfang an war das Ziel, Auswertungen auf Basis der EPRD-Daten der Allgemeinheit transparent zugänglich zu machen. Bereits im Jahr 2016 veröffentlichte das EPRD seinen ersten Jahresbericht und präsentierte, neben der eigenen Registerentwicklung, erstmals deskriptive Daten zu grundlegenden Versorgungsformen in der Hüft- und Knieendoprothetik [3]. Auch wenn der Anteil der im EPRD dokumentierten Versorgungen, bezogen auf die in Deutschland durchgeführten Operationen, damals (Betrachtungszeitraum war das Jahr 2015) nur etwas mehr als ein Drittel ausmachte, war dies der erste Ansatz, die eigene nationale Versorgungsrealität abzubilden [3]. Inzwischen ist der Jahresbericht des EPRD in Deutschland etabliert; seit der Ausgabe 2019 erscheint dieser auch in englischer Sprache und wird national sehr stark wahrgenommen.

### Individuelle Auswertungen für Kliniken und Hersteller

**Figure 1:**
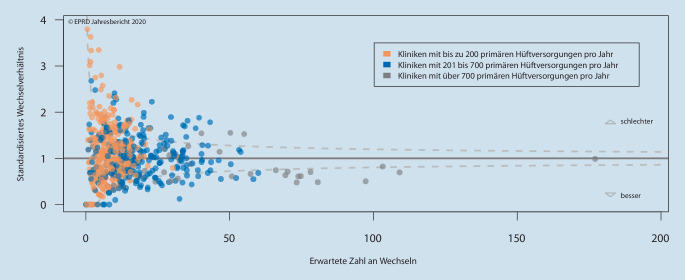

Zusätzlich erhalten teilnehmende Kliniken und Implantathersteller in Deutschland regelmäßig individuelle Auswertungsberichte zu ihren eigenen Ergebnissen bzw. zu denen ihrer Produkte. Jede Klinik, deren Dokumentationen in die Nachverfolgung eingehen, erhält z. B. Analysen zu verschiedenen Versorgungsformen, verwendeten Schafttypen und spezifischen Implantatkombinationen. Außerdem werden die Standzeiten im Vergleich zur jeweiligen Gesamtheit, aber auch in anonymisierter Form mit den Ergebnissen anderer datenliefernder Kliniken verglichen. Dargestellt werden diese Vergleiche u. a. in Form von „funnel plots“ (Trichtergrafiken), die die Anzahl beobachteter vs. erwarteter Wechseloperationen zeigen (Abb. 1). Jede Klinik wird jeweils durch einen Punkt repräsentiert. Wurden für eine Klinik mehr Wechsel beobachtet als erwartet, liegt ihr Punkt über der horizontalen Erwartungslinie, die dem Wert 1 auf der Y‑Achse entspricht. Liegt eine Klinik unter dieser Linie, wurden weniger Wechsel beobachtet als erwartet. Die gestrichelten Linien, die die oberen und unteren Grenzen des 95 %-Konfidenzintervalls trichterförmig kenntlich machen, geben der Darstellung ihren Namen.

Auch die Implantathersteller erhalten neben deskriptiven Übersichten einen Benchmark für ihre Produkte. Verglichen werden hierbei die Ausfallwahrscheinlichkeiten der jeweiligen Implantatkomponenten mit denen aller anderen Implantate derselben Versorgungsform. Diese individuellen Auswertungen sind für Kliniken und Hersteller ein wichtiger Rückkopplungsmechanismus und tragen zur internen Bewertung und damit zu möglichen Qualitätsmaßnahmen bei. Bezüglich der öffentlichen Darstellung spezifischer Auswertungen gibt es im EPRD einen Wermutstropfen. Während zumindest die tabellarische Darstellung implantatspezifischer Ergebnisse seit dem EPRD-Jahresbericht 2017 ein wichtiger Bestandteil der öffentlichen Berichterstattung ist, gibt es bezogen auf die Klinikergebnisse keine entblindete Darstellung [5]. Den Kliniken steht es allerdings frei, ihre eigenen Ergebnisse zu veröffentlichen und diese so der Allgemeinheit und ihren Patienten zugänglich zu machen. Einige Kliniken machen von dieser Möglichkeit bereits Gebrauch.

## Wesentliche Erkenntnisse

**Figure 2:**
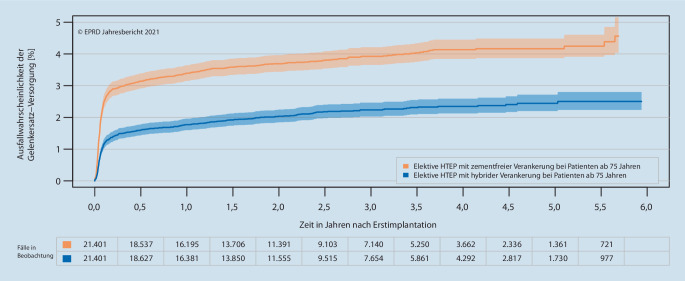

Bereits im zweiten EPRD-Jahresbericht konnten erstmals Standzeitergebnisse bis zu 2 Jahren nach Erstimplantation dargestellt werden. Schon damals waren zwischen den grundlegenden endoprothetischen Versorgungsformen und unterschiedlichen patientenspezifischen Risikoprofilen teilweise deutliche Unterschiede bezogen auf die frühen Ausfallwahrscheinlichkeiten erkennbar. So zeigten beispielsweise Hüfttotalendoprothesen(HTEP)-Versorgungen mit zementiertem Schaft in höheren Altersgruppen signifikant geringere Ausfallwahrscheinlichkeiten als entsprechende Versorgungen mit unzementiertem Schaft [4]. Aber erst mit längerem Betrachtungszeitraum und zunehmender Größe des Datensatzes gelang es im Jahresbericht 2021, unter Berücksichtigung multipler Einflussfaktoren und der Mortalität den „Vorteil“ einer Schaftzementierung bei Patienten im höheren Alter (> 75 Jahre) klar zu zeigen (Abb. 2; [7]).

Aus Rückmeldungen der Ärzteschaft hat das EPRD erfahren, dass u. a. die genannten Ergebnisse aus dem EPRD zu einem kritischeren Umgang mit zementfreien Schaftversorgungen im höheren Lebensalter geführt haben. So würden in vielen Kliniken, insbesondere bei Patienten ab 75 Jahren, jetzt wieder zunehmend zementierte Schäfte verwendet. Für die Gesamtheit primärer HTEPs konnte zuletzt ein leichter Rückgang der komplett zementfreien Implantationen und eine Zunahme von Hybridversorgungen im EPRD beobachtet werden [9]. Ob dies bereits ein Effekt des Umdenkens ist, vermag das Register allerdings zum jetzigen Zeitpunkt noch nicht abzuschätzen.

### Patienten- und versorgerspezifische Einflussfaktoren

**Figure 3:**
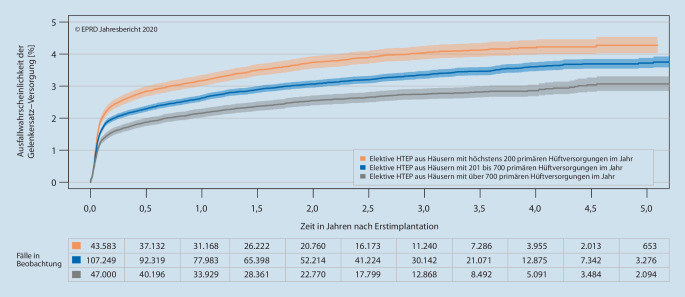

**Figure 4:**
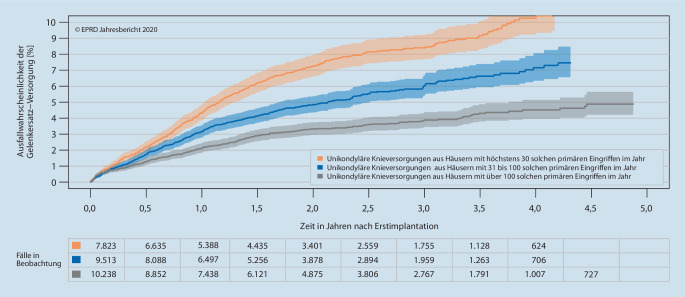

Zu den potenziellen Einflussfaktoren auf das frühe Versorgungsergebnis, die vom EPRD selbst identifiziert werden können, gehören sowohl patientenbezogene Risikofaktoren wie Alter, Geschlecht, Body-Mass-Index (seit 2017), Score der American Society of Anesthesiologists (seit 2020) und das Vorliegen bestimmter Komorbiditäten als auch die „institutionelle Erfahrung“ bezogen auf das Versorgungsvolumen behandelnder Kliniken [4, 6, 8, 20]. Letzteres lässt sich auf Basis der Qualitätsberichte der Kliniken ermitteln [15]. So konnte sowohl für elektive HTEPs (Abb. 3) als auch für Knieendoprothesen, hierbei insbesondere für unikondyläre Versorgungen (Abb. 4), gezeigt werden, dass bei höheren Behandlungszahlen (bezogen auf das Jahr 2018) das Risiko von Wechseleingriffen geringer ausfällt [6].

Diese Tendenz bedeutet nicht, dass Kliniken mit höheren Behandlungsfallzahlen im Einzelfall immer bessere Ergebnisse liefern als Kliniken mit niedrigeren Fallzahlen, was man am Beispiel des „funnel plot“ primärer Hüftversorgungen (Abb. 1) sehen kann. Auch wenn die Kliniken mit mindestens 700 Hüftversorgungen im Jahr überwiegend unter der Erwartungslinie liegen und ein nicht unerheblicher Teil davon sogar unterhalb der unteren Grenze des Konfidenzintervalls, d. h. deutlich bessere Ergebnisse haben als alle anderen, finden sich auch einige dieser Kliniken deutlich oberhalb der Erwartungslinie wieder.

Bei Kliniken mit bis zu 200 Hüftversorgungen und solchen mit 200 bis 700 Hüftversorgungen im Jahr gibt es hingegen ebenfalls einen Anteil deutlich unterhalb der Erwartungslinie. Auch wenn die Einteilung in „groß-, mittel- und kleinvolumige Kliniken“ den Einfluss der daraus abgeleiteten Erfahrung eindrucksvoll darstellt, wäre es dennoch erstrebenswert, auch individuelle Operateurergebnisse berücksichtigen zu können, wie es z. B. seit Jahren im NJR der Fall ist.

### Wissenschaftliche Publikationen

Auch spezifische Design- und Materialeigenschaften der verwendeten Implantate haben einen Einfluss auf die Standzeitergebnisse. Die Herausforderung, mögliche Unterschiede kenntlich zu machen, besteht zum einen darin, dass die implantatbedingten Differenzen deutlich durch die bereits genannten patienten- und versorgerspezifischen Einflussfaktoren überlagert werden und sich darüber hinaus im Idealfall erst nach vielen Jahren der Nachverfolgung zeigen sollten. Daher wurden auf Basis von EPRD-Daten Studien initiiert, deren Design diese Herausforderungen durch spezielle Methoden berücksichtigt und deren Ergebnisse mittlerweile sowohl national als auch zunehmend international in „peer-reviewed journals“ publiziert wurden. So wurden beispielsweise mehrere Studien zum Einfluss spezifischer Gleitpaarungscharakteristika bei HTEPs und Knietotalendoprothesen (KTEPs) analysiert [10, 17–19]. Aus den Ergebnissen ließen sich neben grundlegenden Erkenntnissen auch teilweise Handlungsempfehlungen ableiten. Keramische Gleitpaarungen zeigen bei elektiven HTEPs geringere infektionsbedingte Wechselraten als andere übliche Gleitpaarungstypen [18]. Keramisch beschichtete femorale Gleitflächen bei KTEPs wiederum zeigten keinen Unterschied in den infektionsbedingten Wechselraten zu konventionellen Metalloberflächen, aber dafür tendenziell höhere Inzidenzen aseptischer Wechselgründe [10]. Mit höherer Fallzahl und längerer Betrachtungszeit werden weitergehende Analysen spezifischer Systeme und ihrer Oberflächen hinsichtlich dieser Unterschiede näher betrachten. In einer weiteren Studie zu primären HTEP-Versorgungen deuten die Ergebnisse darauf hin, dass überhöhte Pfanneninserts tendenziell eher bei spezifischen Indikationen eingesetzt werden sollten [17].

## Zugriff auf Daten des EPRD für wissenschaftliche Zwecke

In den Bestrebungen, die Autorenkreise außerhalb des Registers zu erweitern, bietet das EPRD seit nunmehr 3 Jahren den strukturierten Zugriff „Dritter“ auf Daten und Auswertungen über ein Antragsverfahren an. Eine wesentliche Voraussetzung bezüglich der sekundären Datennutzung für wissenschaftliche Zwecke ist, dass die eingereichte Fragestellung auf Basis der EPRD-Daten hinreichend beantwortbar ist. Um dies besser abschätzen zu können, haben potenzielle Antragsteller auf der EPRD-Webseite (https://www.eprd.de/de) nicht nur Zugriff auf die Antragsformulare und Informationen zum Verfahren, sondern auch auf weitere Dokumente, die Aufschluss über den EPRD-Datensatz geben. Zusätzlich können die auf der Webseite verfügbaren Publikationen als Orientierungshilfe dienen. Universitäten und Kliniken in ganz Deutschland nutzen bereits die Möglichkeit des Antragsverfahren mit dem Ziel, auf Basis der EPRD-Daten hochwertige Publikationen zu generieren.

## Auffälligkeitsbewertung im Sinne eines Frühwarnsystems

**Figure 5:**
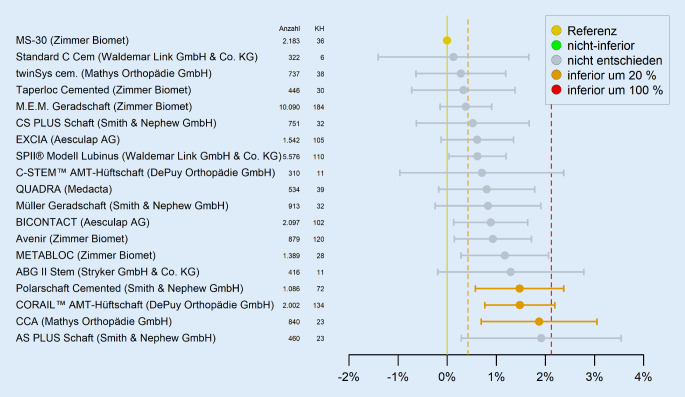

Die Ergebnisdarstellung implantatspezifischer Standzeitergebnisse im EPRD-Jahresbericht erfolgt weitgehend nichtadjustiert. Das bedeutet, dass keine Methoden angewendet werden, um den Einfluss möglicher Störfaktoren, wie z. B. Patientenalter oder Geschlecht, auf das Ergebnis zu minimieren. Der Vorteil dieser ungewichteten Darstellung ist, dass die Gefahr einer Überadjustierung, also einer Adjustierung auf Variablen, die ggf. Teil der Kausalkette sind, vermieden wird. Der Nachteil liegt auf der Hand: Die Bewertung implantatspezifischer Ergebnisse im Sinne eines Benchmarks ist ohne weitergehende Informationen zu den Störfaktoren kaum möglich. Um dem Leser des Jahresberichts bezüglich einzelner Implantatergebnisse dennoch eine bessere Einschätzung zu ermöglichen, werden in den Tabellen zusätzlich der Altersmedian und das Geschlechterverhältnis angegeben. Im Gegensatz zu den neutralen Darstellungen in den EPRD-Jahresberichten können die gleichen Ergebnisse z. B. auch durch eine Inferioritätsbetrachtung mit Bezug zu einer Referenz und in entsprechender Reihung dargestellt werden. In Abb. 5 sind exemplarisch über alle Altersgruppen hinweg die Abweichungen der Ausfallwahrscheinlichkeiten nach 3 Jahren für verschiedene zementierte Schaftsysteme zu einem Referenzschaft aufgezeigt.

Zur besseren Einordnung der Ergebnisse sei an dieser Stelle darauf hingewiesen, dass ein Schaft bereits als ausgefallen gilt, wenn beispielsweise nur Kopf und Insert gewechselt wurden, auch wenn der Schaft weiterhin im Patienten verbleibt. Des Weiteren können sich hinter den verschiedenen Schaftsystemen Versorgungen mit unterschiedlichen Gleitpaarungstypen und Pfannen-Kombinationen verbergen. Wie bereits dargelegt, ist eine Bewertung ohne weitergehende Berücksichtigung potenzieller Einflussfaktoren kaum möglich, sodass die hier dargestellte Reihung keine direkten Rückschlüsse auf die Qualität der betrachteten Implantate zulässt. Daher stellt sich die Frage, wie das Register trotz dieser Einschränkungen potenziell auffällige Standzeiten möglichst frühzeitig und verlässlich identifizieren kann.

Dazu hat das EPRD ein Frühwarnsystem etabliert, bei dem halbjährlich „statistische Ausreißer“ mit erhöhter Revisionsrate ermittelt werden. Unter Berücksichtigung der bekannten Einflussfaktoren werden diese dann in den EPRD-Gremien hinsichtlich ihrer medizinischen Relevanz evaluiert. Betroffene Implantathersteller und Kliniken werden durch das EPRD über relevante Auffälligkeiten informiert und um Stellungnahme gebeten. Die abschließende Bewertung der benannten Auffälligkeit und die Entscheidung über mögliche Konsequenzen obliegen dann der informierten Institution. Da Kliniken und Hersteller ihre eigenen Ergebnisse wie oben dargestellt durch individuelle Standardauswertungen regelmäßig erhalten, ist dieses Frühwarnsystem als eine zusätzliche sensibilisierende Maßnahme zu betrachten.

Noch vor dem Start des EPRD hatten die teils katastrophalen Ergebnisse der Metall-Metall-Gleitpaarungen im australischen Register AOANJRR wesentlich zu einer Marktbereinigung für diese Art von Versorgungen und im Speziellen zu einem Rückruf des ASR-Hüftsystems geführt [2, 12]. Auch wenn solche extremen Implantatprobleme hoffentlich der Vergangenheit angehören, mahnt dieser Fall zu stetiger Wachsamkeit.

Registerdaten haben durch die Medical Device Regulation weiter an Bedeutung gewonnen

Registerdaten haben in den letzten Jahren aufgrund gestiegener regulatorischer Anforderungen an die Überwachung von Implantaten und deren Ergebnisqualität durch die 2017 in Kraft getretene und seit Mai 2021 geltende europäische Verordnung über Medizinprodukte (Medical Device Regulation) weiter an Bedeutung gewonnen. Das Vorliegen genügend klinischer Daten aus Endoprothesenregistern und die Qualität der Ergebnisse haben einen großen Einfluss darauf, ob Implantatkomponenten weiterhin in Verkehr gebracht werden können.

Jedoch deuten die teilweise erheblichen Ergebnisunterschiede zwischen einzelnen Kliniken, die das gleiche Implantatsystem verwenden, darauf hin, dass Auffälligkeiten nicht zwingend dem spezifischen Implantat zuzuordnen sind.

## Mismatch-Identifikation

Eine weitere Möglichkeit, frühzeitig zu warnen, bietet die Mismatch-Detektion des EPRD. Auf Basis der EPRD-Produktklassifikation können im Rahmen der EPRD-Erfassung beispielsweise inkompatible Größen- und Seitenkombinationen von Implantatkomponenten identifiziert werden. Diese Inkompatibilitäten können schwere Komplikationen und großen gesundheitlichen Schaden für den Patienten nach sich ziehen, wenn dadurch die Funktion oder die Standzeit der Versorgung beeinträchtigt wird. Die möglichst frühzeitige Kenntnis eines Mismatch kann im Einzelfall Schaden vom Patienten abwenden. Ob dies gelingt, hängt auch von den klinikinternen Abläufen ab.

Schließt eine Klinik ihre EPRD-Dokumentation für eine Erstimplantation ab, werden die verwendeten Komponenten auf Kompatibilität geprüft [9]. Wird eine Inkompatibilität festgestellt, kommt es zu einer entsprechenden Rückmeldung über die Erfassungssoftware. Ob es sich dabei um einen Dokumentationsfehler oder um einen echten Mismatch-Fall handelt, kann dann in der Klinik überprüft werden. Wird dieser Vorgang bereits perioperativ durchgeführt, kann ein potenzieller Mismatch-Fall ggf. sogar noch intraoperativ verhindert bzw. korrigiert werden. In den allermeisten Fällen werden die Dokumentationen jedoch erst nach der Operation abgeschlossen. Aber auch dann ist eine möglichst frühe Kenntnis darüber, ob ein Mismatch vorliegen könnte, wesentlich für den weiteren Verlauf. Über Rückmeldungen aus einzelnen Kliniken erfuhr das EPRD beispielsweise, dass aufgrund der EPRD-Warnmeldung echte Mismatch-Fälle frühzeitig entdeckt und teilweise noch während des klinischen Aufenthalts revidiert werden konnten oder, wenn es medizinisch vertretbar erschien, der betroffene Patient engmaschiger kontrolliert wurde.

An dieser Stelle muss explizit darauf hingewiesen werden, dass die Mismatch-Detektion die Kliniken zwar unterstützen, aber nicht die Sorgfalt und Prüfung innerhalb der Kliniken ersetzen kann. Zum einen prüft das EPRD nicht auf alle theoretisch möglichen Mismatch-Fälle und zum anderen kann auch nicht gewährleistet werden, dass bei allen der mittlerweile mehr als 68.000 Implantatkomponenten jedes Attribut korrekt hinterlegt wurde. Darüber hinaus gibt es insbesondere in der Knieendoprothetik unterschiedliche herstellerspezifische Größenkompatibilitäten, die keine einheitliche Regelung zulassen. Jedoch ist geplant, ggf. herstellerspezifische Regeln zu implementieren, um auch diese Lücke schrittweise zu schließen.

## Perspektiven aus Sicht des Registers

Das EPRD tritt hinsichtlich der Standzeitanalysen in den kommenden Jahren in eine sehr spannende Phase langfristiger Ergebnisse ein, bei der die Haltbarkeit und das Langzeitverhalten bestimmter Implantatmaterialien und Designeigenschaften im Fokus stehen werden.

Die Patientenbefragung rückt zunehmend ins Blickfeld von Endoprothesenregistern

Unter den aktuellen Rahmenbedingungen und mit knapp 750 datenliefernden Kliniken werden die jährlichen Erfassungszahlen bzw. die Abdeckungsraten im EPRD erwartbar nicht mehr deutlich steigen. Nur auf gesetzlicher Basis wird eine nationale Vollerhebung zu erreichen sein. Daher liegt der Schwerpunkt im EPRD darin, sich qualitativ weiterzuentwickeln. Die Erfahrungen, die in den letzten 10 Jahren im EPRD gesammelt wurden, zeigen, dass hinsichtlich der Erfassung von Parametern, die relevanten Einfluss auf das Versorgungsergebnis haben können, noch weitere Ergänzungen sinnvoll wären. Immer unter der Prämisse der Datensparsamkeit erwägt das EPRD daher die zusätzliche Erfassung operativer Zugänge in der Hüftendoprothetik sowie der Verwendung von Navigation und Robotik in der Knieendoprothetik. Darüber hinaus ist auch die pseudonymisierte Erfassung des Operateurs geplant.

Ein weiterer wichtiger Aspekt, der in den letzten Jahren weltweit zunehmend ins Blickfeld von Endoprothesenregistern gerückt ist, ist die Patientenbefragung. Dazu gibt es mittlerweile eine Vielzahl von standardisierten Befragungsinstrumenten, die „patient-reported outcome measures“ (PROMs). Als Ergänzung zur Erfassung der Implantatstandzeit kann so neben der allgemeinen Lebensqualität auch die Zufriedenheit der Patienten mit dem erreichten Schmerzniveau und der Funktion des endoprothetischen Gelenks gemessen werden, um Wirksamkeit und Leistung endoprothetischer Versorgungen abzuschätzen. In einer kürzlich veröffentlichten Studie der Organisation for Economic Cooperation and Development [16] wurden 13 Endoprothesenregister identifiziert, die bereits PROMs für diese Zwecke nutzen. Als Schmerz- und Funktionsscore wurde in 8 Registern der Oxford Hip Score bzw. in 7 der Oxford Knee Score genutzt. Das EPRD plant für das Jahr 2023 die digitale Erfassung validierter Oxford-Scores im Rahmen eines Probebetriebs über ein eigenes Webportal. Die Befragungslast für Patienten ist dabei mit 12 Fragen überschaubar. Der Aufwand aufseiten der Kliniken soll minimiert und Doppelerfassungen in Kliniken, die bereits entsprechende PROMs erheben, sollen möglichst vermieden werden. Nicht nur Kliniken und Hersteller sollen zukünftig Zugriff auf ihre PROM-Ergebnisse haben und entsprechende Benchmarks erhalten, auch Patienten sollen auf ihre individuellen Ergebnisse zugreifen und sehen können, wie sie im Vergleich zu anderen Patienten z. B. ihres Alters und Geschlechts abschneiden.

## Fazit für die Praxis

- Mit dem EPRD wird die endoprothetische Versorgungslandschaft in Deutschland immer detaillierter abgebildet.
- Ergebnisse aus dem Register tragen dazu bei, bestimmte Versorgungsformen patienten- und implantatbezogen im operativen Alltag zu überdenken und ggf. anzupassen.
- Mit den klinikindividuellen Ergebnissen können datenliefernde Kliniken ihr eigenes Abschneiden kontrollieren und so zur Qualitätsverbesserung in der Endoprothetik beitragen.
- Mit mehr als 2 Mio. Datensätzen ist das EPRD inzwischen weltweit das drittgrößte Register seiner Art.
- Die mit dem NJR harmonisierte Produktdatenbank soll als internationaler Standard eingeführt werden.
- Ab 2023 wird auch die Erfassung von PROMs im EPRD im Rahmen eines Probebetriebs gestartet. Somit wird zukünftig nicht nur die Standzeit der Implantate, sondern auch die Zufriedenheit der Patienten überprüfbar.
- Auch die Erfassung des einzelnen Operateurs ist eine sinnvolle Ergänzung und wird perspektivisch eingeführt werden.

## Abkürzungen

EPRD: Endoprothesenregister Deutschland
HTEP: Hüfttotalendoprothese
KTEP: Knietotalendoprothese
NJR: National Joint Registry
PROMs: „Patient-reported outcome measures“

## References

[1] Blömer W, Steinbrück A, Schröder C (2015). A new universal, standardized implant database for product identification: a unique tool for arthroplasty registries. Arch Orthop Trauma Surg.

[2] Cohen D (2011). Out of joint: The story of the ASR. BMJ.

[3] Grimberg A, Jansson V, Liebs T (2016). Endoprothesenregister Deutschland (EPRD) – Jahresbericht 2015.

[4] Grimberg A, Jansson V, Liebs T (2017). Endoprothesenregister Deutschland (EPRD) – Jahresbericht 2016.

[5] Grimberg A, Jansson V, Liebs T (2018). Endoprothesenregister Deutschland (EPRD) – Jahresbericht 2017.

[6] Grimberg A, Jansson V, Lützner J (2020). Endoprothesenregister Deutschland (EPRD) – Jahresbericht 2020.

[7] Grimberg A, Jansson V, Lützner J (2021). Endoprothesenregister Deutschland (EPRD) – Jahresbericht 2021.

[8] Grimberg A, Jansson V, Melsheimer O (2019). Endoprothesenregister Deutschland (EPRD) – Jahresbericht 2019.

[9] Grimberg A, Lützner J, Melsheimer O (2022). Endoprothesenregister Deutschland (EPRD) – Jahresbericht 2022.

[10] Grimberg AW, Grupp TM, Elliott J (2021). Ceramic coating in cemented primary total knee arthroplasty is not associated with decreased risk of revision due to early prosthetic joint infection. J Arthroplasty.

[11] Grothaus F, Hassenpflug J, Jansson V (2015). Endoprothesenregister Deutschland (EPRD) – Statusbericht 2014.

[12] https://www.jnjmedtech.com/en-US/depuy-synthes/asr-recall. Zugegriffen: 1. Dez. 2022

[13] https://www.eprd.de/fileadmin/user_upload/Dateien/Pressemitteilungen/EPRD-PM-10JahreEPRD-Datenauswertung-ZweiMillionenGelenkersatzoperationen_2022-03-02_F.pdf. Zugegriffen: 1. Dez. 2022

[14] https://www.vdek.com/presse/daten.html. Zugegriffen: 1. Dez. 2022

[15] Iqtig (2019). IQTIG – Institut für Qualitätssicherung und Transparenz im Gesundheitswesen: Qualitätsreport 2019.

[16] Kendir C, Bienassis KD, Slawomirski L (2022). International assessment of the use and results of patient-reported outcome measures for hip and knee replacement surgery.

[17] Krull P, Steinbrück A, Grimberg AW (2022). Modified acetabular component liner designs are not superior to standard liners at reducing the risk of revision : an analysis of 151,096 cementless total hip arthroplasties from the German Arthroplasty Registry. Bone Joint J.

[18] Renner L, Perka C, Melsheimer O (2021). Ceramic-on-ceramic bearing in total hip arthroplasty reduces the risk for revision for periprosthetic joint infection compared to ceramic-on-polyethylene: a matched analysis of 118,753 cementless THA based on the German Arthroplasty Registry. J Clin Med.

[19] Steinbrück A, Grimberg AW, Elliott J (2021). Short versus conventional stem in cementless total hip arthroplasty: An evidence-based approach with registry data of mid-term survival. Orthopäde.

[20] Steinbrück A, Melsheimer O, Grimberg A (2020). Einfluss der institutionellen Erfahrung auf die Ergebnisse in Hüft- und Knietotalendoprothetik. Orthopäde.

